# Topical cell-free conditioned media harvested from adipose tissue-derived stem cells promote recovery from corneal epithelial defects caused by chemical burns

**DOI:** 10.1038/s41598-020-69020-z

**Published:** 2020-07-24

**Authors:** Gae Won Park, Jeonghoon Heo, Jung Youb Kang, Ji Won Yang, Jong Sik Kim, Ki Dong Kwon, Byung Chul Yu, Sang Joon Lee

**Affiliations:** 1 Stem Bank Company, Busan, Korea; 2 Department of Molecular Biology and Immunology, College of Medicine, Kosin University, Busan, Korea; 3 The BGN Eye Hospital, Busan, Korea; 4 Department of Ophthalmology, College of Medicine, Kosin University, #34 Amnam-dong, Suh-ku, Busan, 602-702 Korea; 5 Department of Anatomy, College of Medicine, Kosin University, Busan, Korea; 6 Department of Preventive Medicine, College of Medicine, Kosin University, Busan, Korea

**Keywords:** Translational research, Mesenchymal stem cells

## Abstract

Corneal chemical burns can lead to blindness following serious complications. As most of these complications are caused by failure of reepithelization during the acute phase, treatment at this stage is critical. Although there have been some studies on corneal injury recovery using adipose tissue-derived stem cells (ADSCs), none has reported the effect of topical cell-free conditioned culture media (CM) derived from ADSCs on corneal epithelial regeneration. Here, the best conditions for CM were selected and used for in vitro and in vivo experiments. Corneal burn in rats was induced using 100% alcohol. The chosen CM was administered to corneal burn rats (CM-treated [CT] group) four times a day for three days and this group was compared with the normal control and corneal burn (CB) groups. Biomicroscopic fluorescence images and the actual physical corneas were taken over time and used for analysis. mRNA levels of hepatocyte growth factor and epidermal growth factor (EGF) were significantly increased, whereas those of vascular endothelial growth factor, interleukin (IL)-1β, IL-6, IL-10, and matrix metalloproteinase-9 were significantly decreased in the CT group compared with those in the CB group. The numbers of proliferating cell nuclear antigen- and zonular occludens-1-positive cells in the CT group were significantly higher than those in the CB group. The macrophage-infiltrating corneas in the CT group expressed significantly more of the M2 marker arginase than corneas in the CB group. Optimal CM (× 0.5 concentration) treatment significantly accelerated the migration of corneal epithelial cells and induced upregulation of the expression of IL-6, EGF, and C-X-C chemokine receptor type 4 mRNAs. Overall, in this study, topical administration of cell-free CM promoted regeneration of the corneal epithelium after induction of chemical burns.

## Introduction

Corneal chemical burns are an ophthalmic emergency that can lead to blindness and require immediate evaluation and treatment. Serious complications of chemical injury include slow epithelization, persistent epithelial defects, corneal melting and perforation, corneal opacity, and neovascularization. As most of these complications are caused by failure of reepithelization in the acute phase, treatment at this stage is critical^1,2^. Clinically, the main focus of acute phase therapy is to control inflammation and quickly recover the corneal epithelium. Several new steroid drugs have been developed, but complications such as cataracts, glaucoma, and delayed epithelization can occur from their long-term usage^3,4^. Amniotic membrane transplantation and limbal stem cell transplantation are also fraught with certain problems, including low utilization rate and immune response^5^. Therefore, new therapies must be explored to overcome these issues.

Mesenchymal stem cells (MSCs) are multipotent cell types that were initially isolated from bone marrow (BM) and subsequently separated from other tissues, including fat^6^, cardiac tissue^7^, cord blood^8^, and oral tissue^9^. In particular, adipose tissue-derived stem cells (ADSCs) are abundant in the human body and have multiple differentiation potentials, making them a potential material for wound healing and tissue engineering with low risk in terms of cell acquisition and easy processing^10^. ADSCs share many similar biological characteristics with BM-derived MSCs (BMSCs), such as immunophenotype, multipotent differentiation, cytokine secretion profile, and immunomodulatory effects^11,12^. However, depending on the tissue source, donor, isolation, and culture protocol, the properties of MSCs may change slightly^12–14^. Despite these minor differences, ADSCs seem to have clinical advantages over BMSCs or the other sources given their convenience of harvesting and abundance of sources.

Although MSCs were expected to improve refractory diseases by differentiating into various tissue cells^15,16^, many studies have failed to achieve the anticipated results based on low engraftment rates^17^. In recent years, paradigm shifts, such as the use of cell-free therapies with stem cell-secreted growth factors, exosomes, or cytokines, have been seen^18^. MSCs help repair damaged cells and tissues in various ways, such as differentiation and proliferation through paracrine signaling, which is known to have a beneficial effect on wound healing by reducing inflammation and promoting angiogenesis to induce cell migration and proliferation^19,20^.

In this regard, conditioned culture media (CM) has potential as an ophthalmic topical drop to improve recovery of the epithelium of the ocular surface. In addition, analysis of CM from BMSCs revealed that they secrete mediators for corneal epithelial repair, including vascular endothelial growth factor (VEGF), platelet-derived growth factor (PDGF), basic fibroblast growth factor (bFGF), epidermal growth factor (EGF), keratinocyte growth factor (KGF), transforming growth factor β (TGFβ), and hepatocyte growth factor (HGF)^21–23^.

MSCs have been garnering interest within the field of corneal regeneration^8,24,25^. In particular, BMSCs or their CM inhibit inflammation and neovascularization, promoting corneal recovery when applied to chemically damaged corneas of rats^24–28^. Although there have been some investigations of corneal injury recovery using ADSCs, none have reported the effect of topical CM derived from ADSCs on corneal epithelial regeneration and the pertinent mechanism of action^29,30^. The purpose of this study was to evaluate whether serum-free CM harvested from ADSCs is effective in corneal epithelial healing after induction of chemical injury and identify factors that respond to CM in terms of corneal regeneration.

## Results

### Characteristics of rat ADSCs

**Figure 1:**
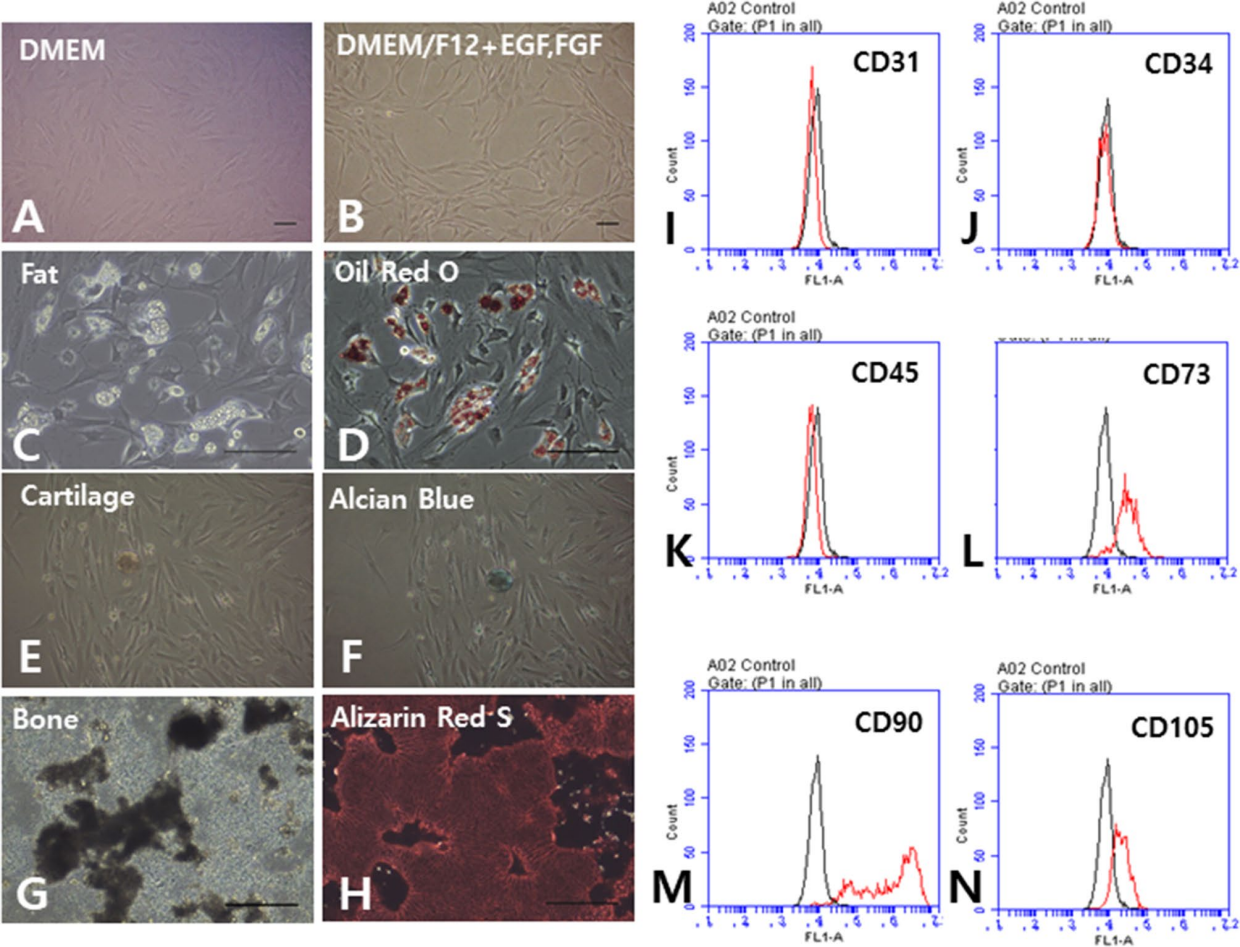
Characteristics of cultured rat adipose tissue-derived stem cells (ADSCs). Use of DMEM without growth factors resulted in a relatively large and round epithelial morphology of rat ADSCs (**A**), whereas use of DMEM/F12 medium with growth factors resulted in relatively small cells with a fibroblast-like spindle shape (**B**). After 3 weeks of mesenchymal differentiation induction, various lineage differentiations, including fat (**C**), cartilage (**E**), and bone (**G**), was observed, which were stained with Oil Red O (**D**), Alcian Blue (**F**), and Alizarin Red S (**H**), respectively. Surface markers were examined using flow cytometry to define mesenchymal-origin stem cells. Subcultured rat ADSCs were negative for CD31 (**I**), CD34 (**J**), and CD45 (**K**), but were 32.6%, 86.5%, and 24.2% positive for CD73 (**L**), CD90 (**M**), and CD105 (**N**), respectively. Scale bar, 100 μm; ADSCs, adipose tissue-derived stem cells; Scale bar, 100 μm.

The primary cultured cells were initially round and cuboidal in shape and fused to fusiform-like fibroblasts. Cell morphology varied according to culture media and the added growth factors. Culture in DMEM without growth factors resulted in a relatively large and round rat ADSC epithelial morphology, whereas use of DMEM/F12 medium with growth factors resulted in relatively small, spindle-shaped cells similar to fibroblasts (Fig. 1A,B). In other words, rat ADSCs were different in shape depending on the type of culture medium and growth factors. After 3 weeks of mesenchymal differentiation, various lineage differentiations, including fat (Fig. 1C), cartilage (Fig. 1E), and bone (Fig. 1G), were observed, which were stained with Oil Red O (Fig. 1D), Alcian Blue (Fig. 1F), and Alizarin Red S (Fig. 1H), respectively. Subsequently, surface markers were examined using flow cytometry to determine mesenchymal-origin stem cells. Subcultured rat ADSCs were 32.6%, 86.5%, and 24.2% positive for the MSC markers CD73, CD90, and CD105, respectively, and negative for the endothelial cell marker CD31, hematopoietic progenitor marker CD34, and panleukocyte marker CD45 (Fig. 1I–N). The expression rate of CD73, CD90, and CD105 was lower than that in human MSCs presented by the International Society of Cell therapy (ISCT)^31^. However, this has been reported a number of times in rats because of different species, isolation, culture methods, and location of fat^12,32,33^.

### Proliferation and migration rate of rat ADSCs according to culture medium composition

**Figure 2:**
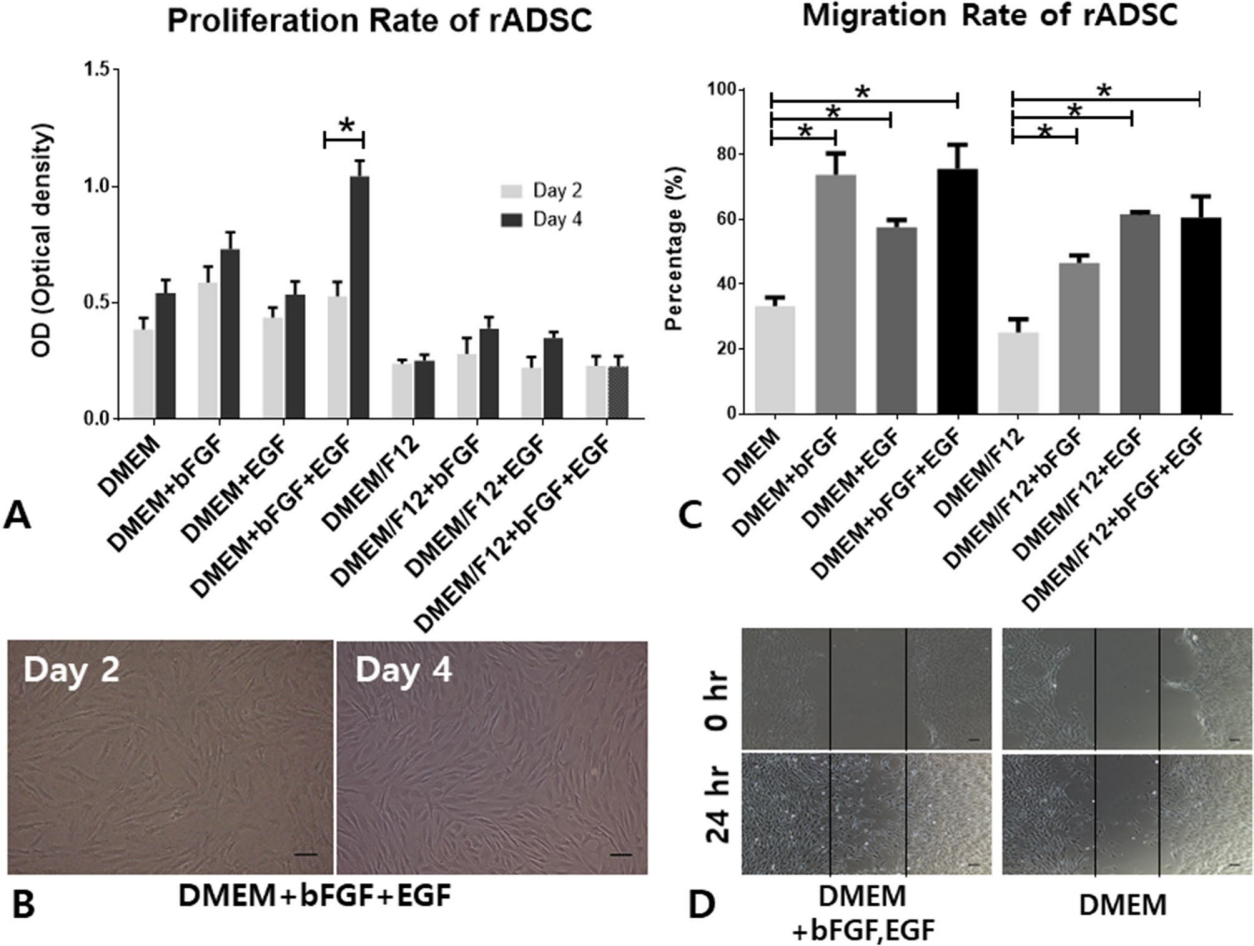
Proliferation and migration rate of rat ADSCs depending on culture media. Eight kinds of culture media were tested for proliferation and migration rates. Proliferation rates were measured using an MTT assay on the second (baseline) and fourth day after implantation of 1 × 10^4 ^cells/cm^2^ in a 24-well plate. Proliferation rate was significantly higher at 4 days compared with baseline in DMEM + bFGF + EGF (*p* = 0.0043, **A**,**B**). Migration rates were measured using the scratch-assay method (**C**,**D**). After confluence of the transplanted cells, the plates were scratched using a pipette tip and scratch gaps between 0 and 24 h were compared. The equation for the percentage of migration was (100-wound area at 24 h)/wound area at 0 h × 100. Scale bar, 100 μm; ADSCs, adipose tissue-derived stem cells; OD, optical density; scale bar, 100 μm.

Eight kinds of culture media—10% DMEM, 10% DMEM + bFGF, 10% DMEM + EGF, 10% DMEM + bFGF + EGF, 10% DMEM/F12, DMEM/F12 + bFGF, DMEM/F12 + EGF, and DMEM/F12 + bFGF + EGF—were tested for proliferation and migration rates of rat ADSCs. The proliferation rate for DMEM + bFGF + EGF was higher than those for the other culture media (*p* = 0.0043, Fig. 2A). The representative pictures for DMEM + bFGF + EGF on the second and fourth days of the proliferation assay are shown in Fig. 2B. Interestingly, DMEM-based media exhibited a better proliferation ability compared to DMEM/F12-based media. Migration rates of rat ADSCs were measured using the scratch-assay method. Migration rates were significantly higher in media containing growth factors irrespective of DMEM or DMEM/F12 (Fig. 2C). The representative pictures for DMEM with cytokines at 0 and 24 h show enhanced migration ability of rat ADSCs compared with the pictures for DMEM (Fig. 2D).

### Profile of cytokine secretion from rat ADSCs by culture medium composition

**Figure 3:**
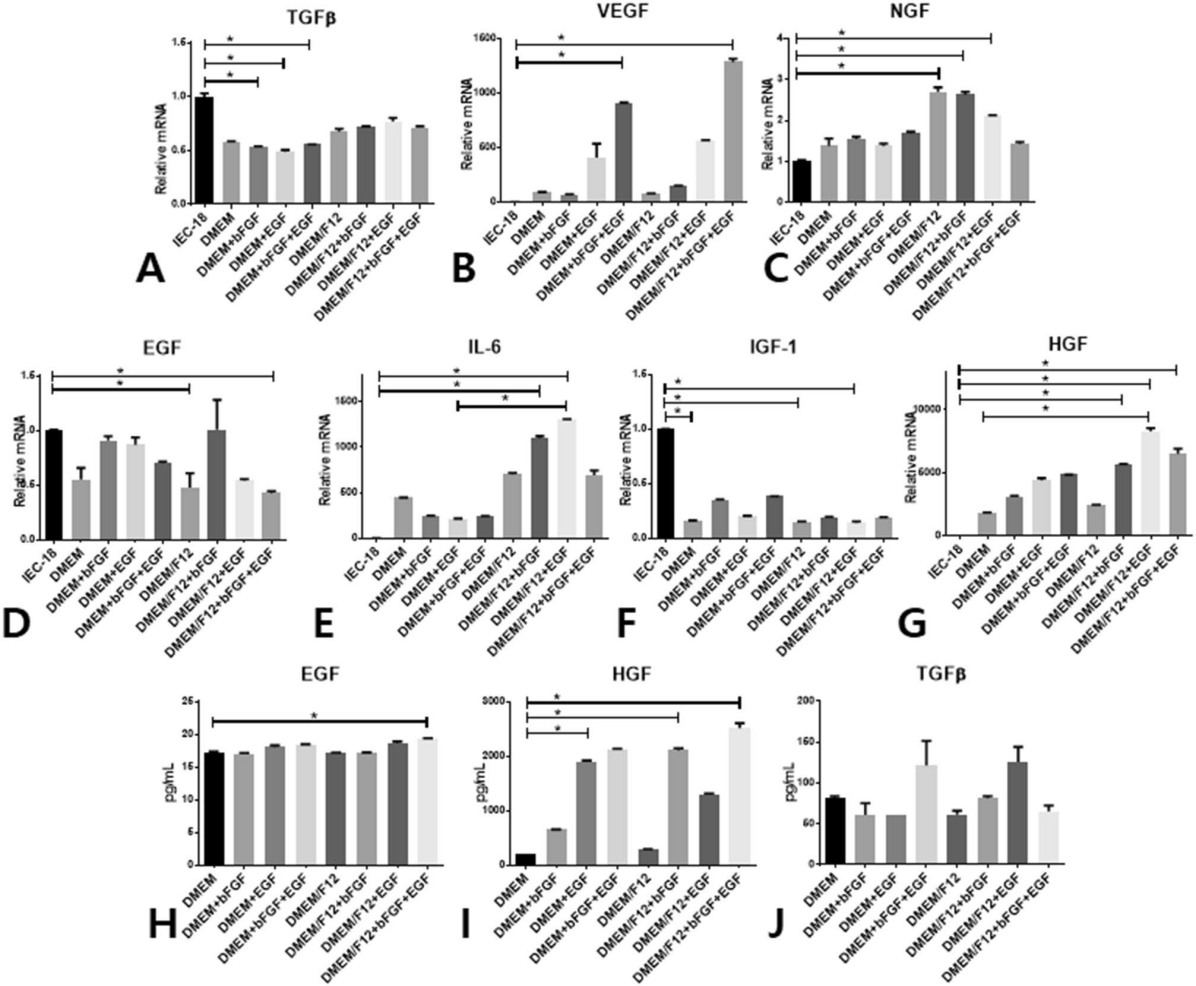
Secretion of cytokines and growth factors from rat ADSCs depending on culture media. Confluent P5 rat ADSCs cultured in eight kinds of culture media were transferred into serum-free media and cultured for 24 h, and then the culture media and ADSCs were harvested for gene and protein analysis. Total RNA was extracted for real-time PCR. mRNA levels for TGFβ, VEGF, NGF, EGF, IL-6, IGF-1, and HGF were compared among the eight groups (**A**–**G**). The amounts of EGF, HGF, and TGFβ were measured using ELISA (**H**–**J**). IEC-18, a rat intestinal epithelial cell line, was used as a control. *p < 0.05; ELISA, enzyme-linked immunosorbent assay; ADSC, adipose tissue-derived stem cells; TGFβ, transforming growth factor β; VEGF, vascular endothelial growth factor; NGF, nerve growth factor; EGF, epidermal growth factor; IL-6, interleukin-6; IGF-1, insulin-like growth factor-1; HGF, hepatocyte growth factor.

Cultured rat ADSCs secreted various amounts of growth factors and cytokines depending on the eight kinds of culture media. The expression of VEGF, NGF, IL-6, and HGF mRNAs was higher than that in the control; VEGF expression was higher in DMEM + EGF and DMEM/F12 + bFGF + EGF; NGF in DMEM/F12, DMEM/F12 + bFGF, and DMEM/F12 + bFGF + EGF, IL-6 in DMEM/F12 + bFGF, DMEM/F12 + EGF; and HGF in DMEM/F12 + bFGF, DMEM/F12 + EGF, and DMEM/F12 + bFGF + EGF (Fig. 3B,C,E,G). IEC-18 intestinal mucosal epithelial cells were used as the epithelial control cell line. TGFß, EGF, and ICF-1 mRNAs were expressed in lower quantities than those in the control (Fig. 3A,D,F). The protein levels of EGF, HGF, and TGFß in CM harvested from rat ADSCs cultured in the eight kinds of culture media were evaluated using enzyme-linked immunosorbent assay (ELISA; Fig. 3H–J). The CM of rat ADSCs cultured in DMEM/F12 + EGF + bFGF contained a larger amount of HGF compared with the other media (*p* = 0.0021). Interestingly, HGF mRNA synthesis was highest in DMEM/12 + EGF but HGF protein secretion was greatest in DMEM/12 + EGF + FGF. Thereafter, DMEM/F12 with EGF and bFGF were regarded as the optimal medium for culturing rat ADSCs before harvesting CM.

### Regeneration of corneal epithelial defects by topical CM administration

**Figure 4:**
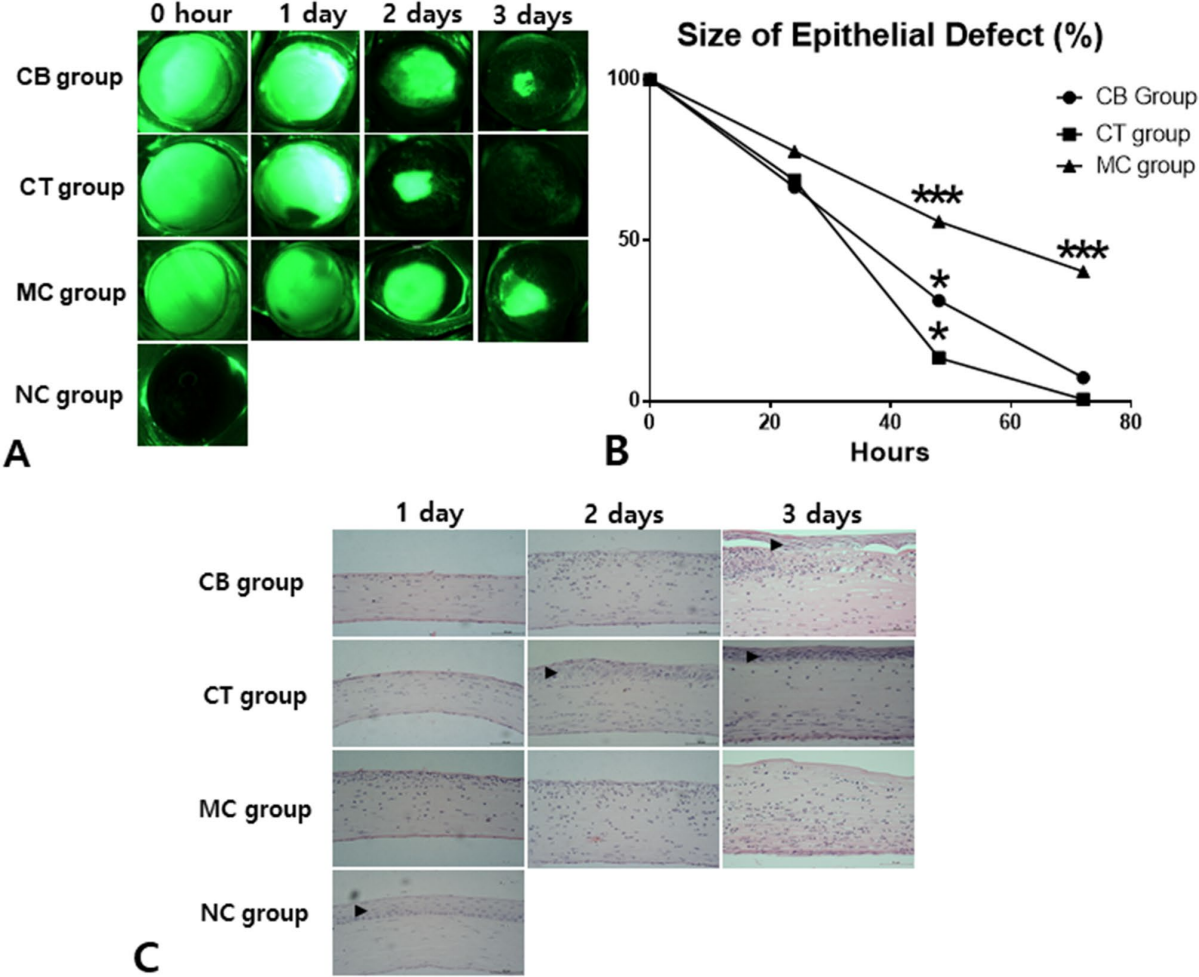
Corneal epithelial recovery after topical administration of conditioned media (CM) harvested from rat ADSCs. Fluorescein-stained corneal pictures were taken at 0 h, 12 h, 1 day, 2 days, and 3 days after induction of chemical burn. The epithelial defect was traced and the area was measured with Image J. Significantly faster recovery from epithelial defect was observed in the CM group from the second day of injury onwards compared with the corneal burn (CB), normal control (NC) and media control (MC) groups (**A**,**B**). The epithelial differentiation in the CM group was better than that in the CB group in terms of HE staining of the cornea at 3 days (**C**). The epithelium layer is marked by a black arrowhead. ADSC, adipose tissue-derived stem cells; CM, conditioned media; scale bar, 1 mm and 50 μm, (**A**) and (**B**), respectively.

Pictures of fluorescein-stained corneas of the CB, CT, MC, and NC groups were taken at 0 h, 12 h, 1 day, 2 days, and 3 days after induction of chemical burn. Epithelial defects of the cornea were stained using fluorescein dye. No fluorescein stain was observed in the NC group. The size of the epithelial defects were compared with each other in the CB, CT, and MC groups (Fig. 4A,B), and it decreased rapidly in CT group compared with the other groups from the second day onwards. Representative pictures are shown in Fig. 4A. On the second day after injury, the percentage of epithelial defects was 13.60 ± 8.30% in the CT group, 31.38 ± 20.02% in the CB group, and 55.93 ± 2.60% in the MC group (*p* < 0.0001, Fig. 4B). Interestingly, the MC group showed delayed epithelial recovery compared to the CB and CT groups. On the third day, epithelial recovery of the CT group was nearly complete, but epithelial defects of the CB and MC groups was observed. The percentage of epithelial defects was 0.64 ± 0.66% in the CT group, 7.40 ± 7.02% in the CB group, and 40.40 ± 12.27% in the MC group.

The degree of differentiation of the corneal epithelium is depicted in Fig. 4C. A well-differentiated corneal epithelium was observed in the CT group from the second day onwards. In this regard, the topical CM had an effect on corneal epithelial differentiation as well as acceleration of corneal epithelial recovery.

### Sequential mRNA expression profiles in corneas

**Figure 5:**
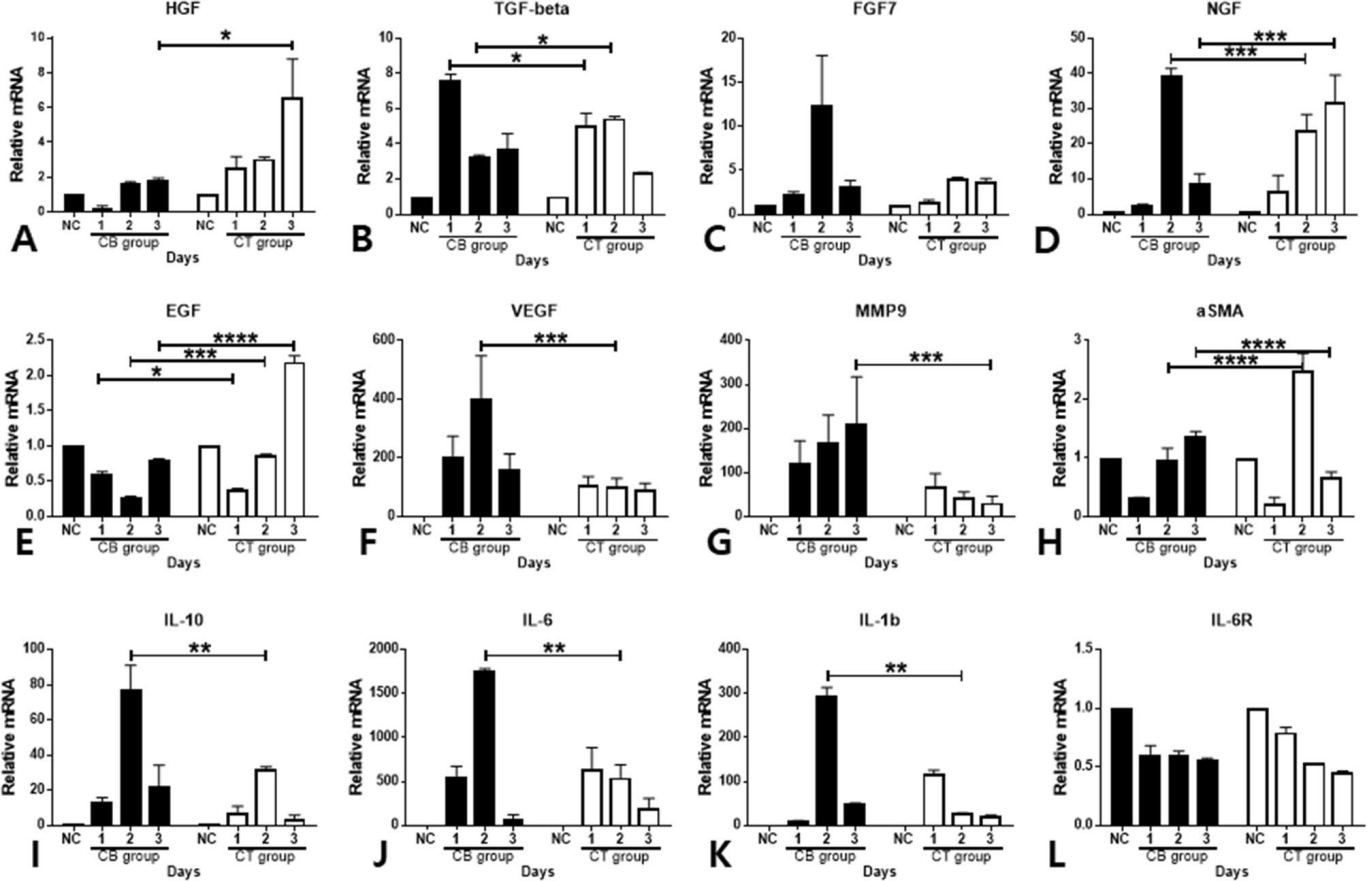
Sequential mRNA expression of HGF, TGFβ, FGF7, NGF, EGF, VEGF, MMP9, aSMA, IL-10, IL-6, IL-1β, and IL-6R in CM, CB, and NC groups (**A**–**L**). The corneas were harvested 0 days, 1 day, 2 days, and 3 days after induction of chemical burn. CM was administered to the cornea of the CM group four times a day topically. In the CB group, the corneal epithelial defect was left without any intervention. HGF, hepatocyte growth factor; TGFβ, transforming growth factor β; FGF7, fibroblast growth factor 7; NGF, nerve growth factor; EGF, epidermal growth factor; VEGF, vascular endothelial growth factor; MMP9, Matrix metallopeptidase-9; IL-10, interleukin 10; IL-6, interleukin-6; IL-1, interleukin-1; αSMA, alpha smooth muscle actin.

Corneas were harvested at 0 h, 1 day, 2 days, and 3 days after induction of chemical burn to analyze mRNA levels. HGF mRNA was significantly overexpressed on the third day after injury in the CT group, which was statistically significant (*p* < 0.05, Fig. 5A). TGFβ mRNA was elevated in both groups, but a significant increase was observed in the CB group on the first day after injury and in the CT group on the second day after injury (Fig. 5B). There was no significant difference in FGF7 mRNA level between the two groups (Fig. 5C). NGF mRNA level was significantly elevated in the CB group on the second day after injury and in the CT group on the third day after injury (*p* < 0.0005, Fig. 5D). EGF mRNA level was significantly increased in the CB group on the first day after injury (*p* < 0.05) but significantly elevated on the second (*p* < 0.0005) and third (*p* < 0.00005) days after injury in the CT group (Fig. 5E). VEGF mRNA level was significantly decreased in the CT group on the second day after injury compared to the CB group (*p* < 0.00005, Fig. 5F). In the case of MMP-9 mRNA level, a significant reduction was observed in the CT group compared with that in the CB group on the third day after injury (*p* < 0.05, Fig. 5G). Compared with that in the CB group, αSMA mRNA level in the CT group was increased on the second day (*p* < 0.00005) after injury but decreased on the third day (*p* < 0.00005, Fig. 5H). mRNA levels of IL-10, IL-6, and IL-1β were significantly decreased in the CT group compared with those in the CB group on the second day after injury (*p* < 0.0005, Fig. 5L–K). There was no significant change in IL-6R mRNA level (Fig. 5L). Based on the data, topical CM administration was considered to upregulate the expression of EGF and HGF mRNAs while downregulating the expression of VEGF, MMP9, IL-10, IL-6, and IL-1β mRNAs in the corneas of the CT group. TGFβ and NGF mRNAs in the CT and CB groups were up- and downregulated depending on the time points.

### The effect of CM on corneal epithelial regeneration

**Figure 6:**
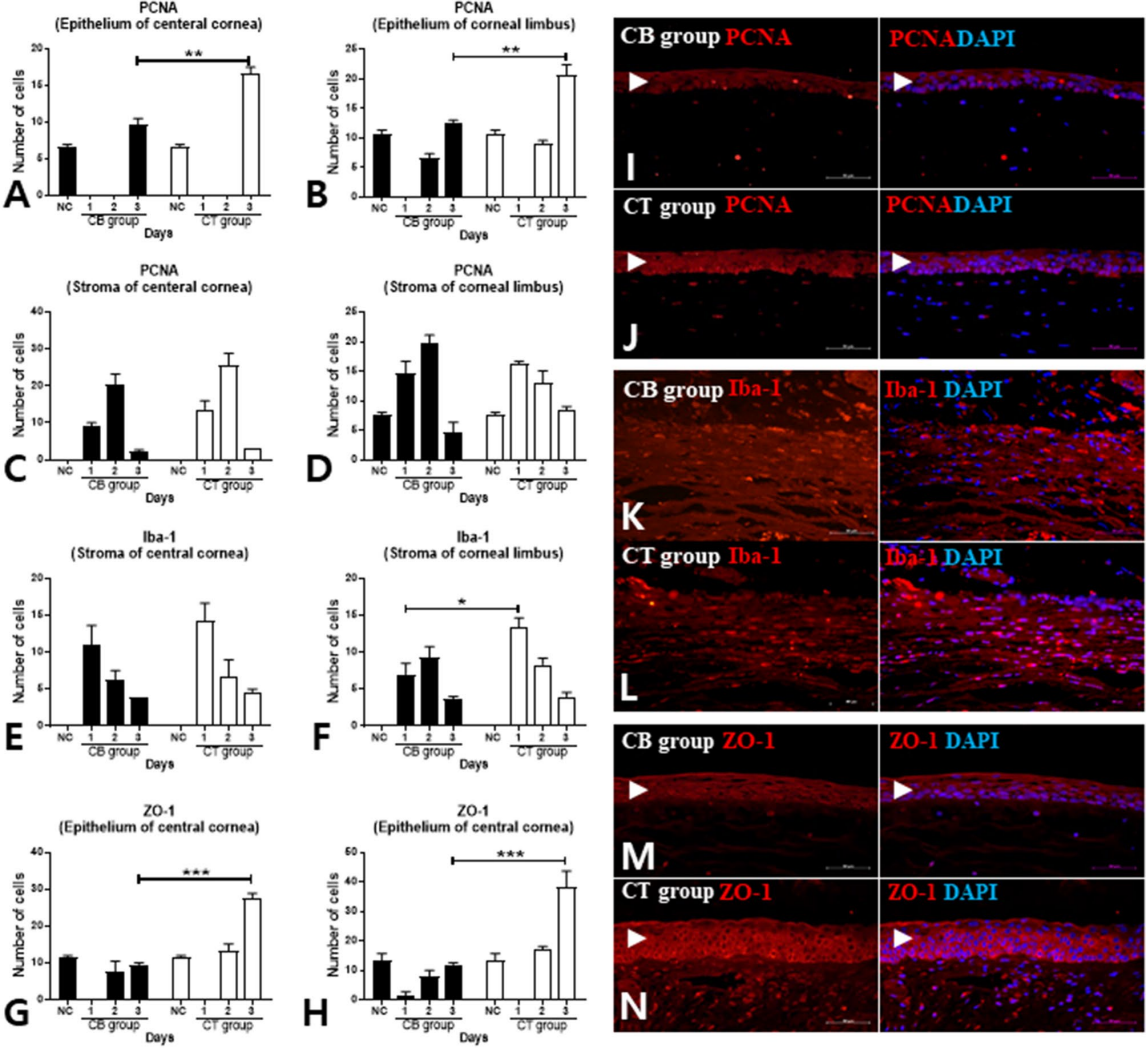
Immunohistochemistry of corneas harvested from the CM, CB, and NC groups. Paraffin section slides of both groups were stained for PCNA (**A**–**D**), Iba-1 (**E**,**F**), or ZO-1 (**G**,**H**) and with DAPI. Cells stained with antibodies and DAPI were counted and compared for each group. The representative immunohistochemically stained pictures of PCNA are shown in (**I**) and (**J**), which were stained at the corneal center of the CB and CM groups, 3 days after induction of chemical burn, respectively. The representative images of Iba-1 are shown in (**K**) and (**L**) of CB and CM groups in the limbal area one day after induction of chemical burn, respectively. The representative pictures of ZO-1 on the third day are shown in M and N of the central epithelium of the CB and CM groups, respectively. The epithelium layer is marked by a black arrowhead. PCNA, proliferating nuclear antigen; Iba-1, ionized calcium binding adaptor molecule-1; ZO-1, zona occludens-1; scale bar, 100 μm.

Immunohistochemistry for PCNA, Iba-1, and ZO-1 was performed, and the results compared between groups. The number of PCNA-positive cells in both the central and peripheral (limbal) corneal epithelium on the third day after induction of chemical burn was significantly higher in the CT group than in the CB group (Fig. 6A,B,I,J). However, the number of PCNA-positive cells in the stroma was similar between the two groups (Fig. 6C,D). The numbers of Iba-1-positive cells in both the central and peripheral (limbal) corneal stroma were similar in both groups on the second and third days after corneal burn; however, the number of Iba-1-positive cells in the limbus of the CT group on the first day was significantly greater than that of the other groups (Fig. 6E,F,K,L). The number of ZO-1-positive cells in both the central and peripheral (limbal) corneal epithelium was significantly higher in the CT group than in the CB group on the third day after induction of chemical burn (Fig. 6G,H,M,N). Topical CM had effects on the proliferation and differentiation of the corneal epithelium.

### CM induces migration and proliferation in human-cultured corneal epithelial cells (HCECs)

**Figure 7:**
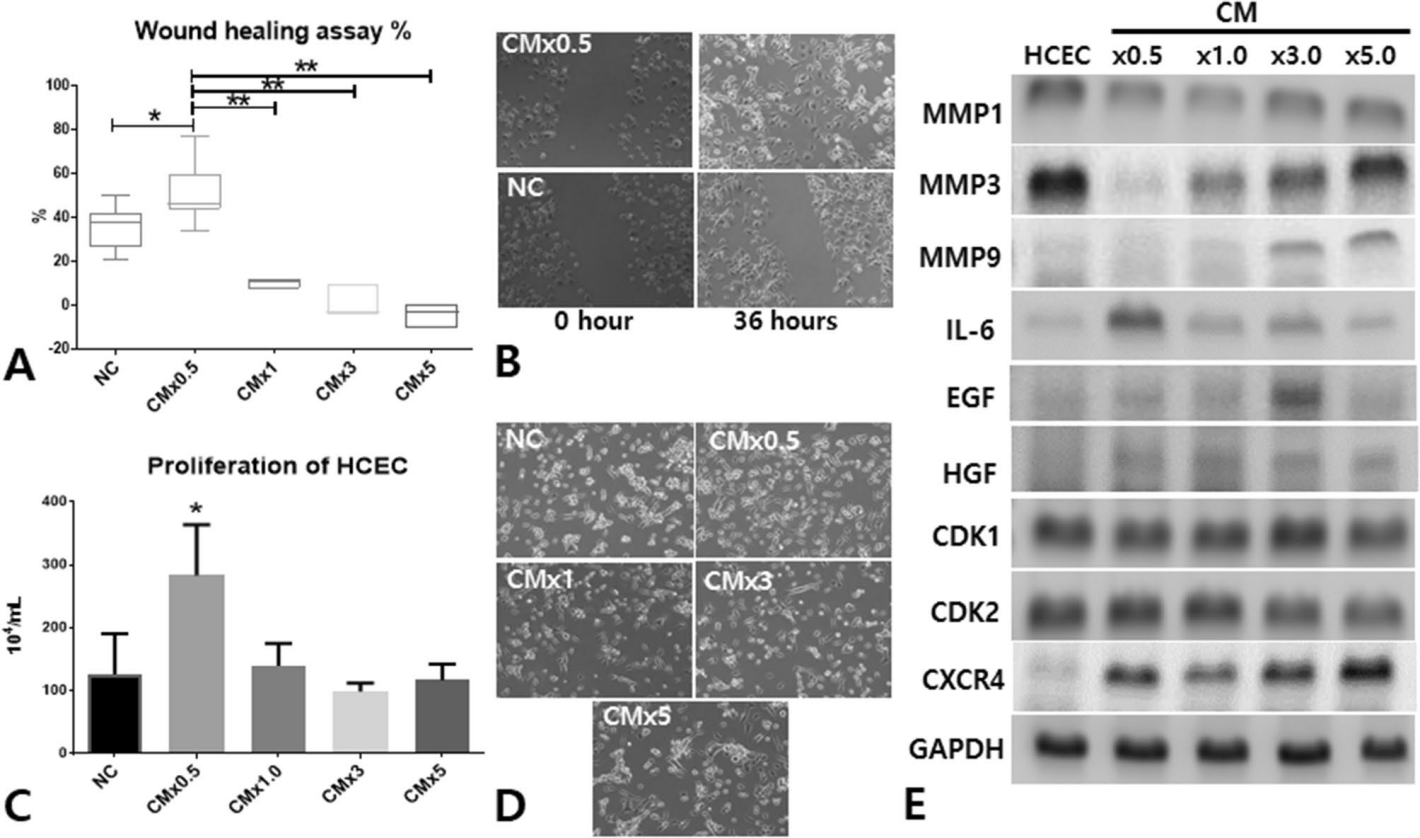
Wound healing and proliferation assay for HCECs depending on CM concentrations (× 0.5, × 1.0, × 3.0, × 5.0). Culture media for the HCEC migration assay was composed of HCEC medium containing supplements with various concentrations of CM (× 0.5, × 1.0, × 3.0, × 5.0) derived from the ADSC culture. After the transplanted cells reached confluence, the plates were scratched using a pipette tip and scratch gaps compared between the 0 h and 48 h time points (**A**,**B**). The equation for the percentage of migration was (100-wound area at 48 h/wound area at 0 h) × 100. To measure the proliferative capacity of the cells, the degree of cell viability was measured using an MTT [3-(4,5-dimethylthiazol-2yl)-2,5-diphenyltetrazoliumbromide] assay at 540 nm (**C**,**D**). CM × 0.5 more potently induced proliferation of HCECs over the other groups. After the migration and proliferation assay, HCECs were harvested and mRNAs for migration-related genes and epithelial growth cytokines were analyzed using PCR (**E**). (**B**) and (**D**), 100X magnification; MMP, matrix metallopeptidase; IL-6, interleukin-6; EGF, epithelial growth factor; HGF, hepatocyte growth factor; CDK, cyclin-dependent kinase; CXCR4, C-X-C chemokine receptor type 4; GAPDH, glyceraldehyde 3-phosphate dehydrogenase.

In order to determine the migration efficiency of CM-treated HCECs, an in vitro scratch-inducing cell migration assay was conducted in various concentrations of CM (× 0.5, × 1.0, × 3.0, × 5.0). Among the tested CM concentrations, CM × 0.5 treatment significantly increased migration efficiency and proliferation of HCECs (Fig. 7A–D). Known migration-associated transcription factors CXCR4 and IL-6 were overexpressed in CM-treated HCECs (Fig. 7E). Interestingly, IL-6 was only upregulated at CM × 0.5. Otherwise, MMP1, MMP3, and MMP9 were not upregulated in CM-treated HCECs. EGF and HGF, necessary growth factors for corneal epithelial regeneration, were overexpressed in CM-treated HCECs. Notably, the upregulation of EGF and HGF corroborated what we observed for the mRNAs of the CM-treated cornea. In addition, CM × 0.5 induced proliferation of HCECs over the other groups, and CDK1 and CDK2 were not involved in proliferation (Fig. 7E). The in vitro migration and proliferation assays for HCECs showed that relatively low concentrations of CM might be more optimal for HCECs in terms of proliferation and migration compared to other high concentrations of CM.

## Discussion

In the present study, the effect of topical CM on corneal epithelial healing was evaluated using in vivo and in vitro experiments. CM promoted re-epithelization of the cornea denuded by chemical burn through involvement of proliferation, migration, and differentiation of corneal epithelial cells. Synthesis of EGF and HGF were upregulated in CM-treated corneas. Based on the data, topical CM eye drops can be a treatment option for corneal epithelial damage. However, the optimal concentration of CM as a topical drop should be evaluated for clinical application.

We investigated which types of media were most suitable as eye drops to regenerate the corneal epithelium. VEGF, IL-6, and HGF were abundant in DMEM/F12 with EGF, bFGF, or both, more so than in DMEM or IEC-18 cultured soup. Kilroy et al. showed that EGF (10 ng/10^6 ^cells) elevated HGF secretion in undifferentiated human ADSCs^21^. However, the specific culture conditions of ADSCs required to harvest key cytokines involved in corneal epithelial regeneration in ADSCs were not evaluated in previous literature. We observed that DMEM/F12 with EGF and bFGF may represent the optimal condition to generate CM as a topical eye drop.

ADSCs secrete putative paracrine factors. Among them, several cytokines, including HGF, IL-6, NGF, EGF, VEGF, IGF-1, and TGFβ, are key factors involved in corneal epithelial regeneration^34^.

Most of the previous studies using MSCs for corneal epithelial regeneration used BM as the source of MSCs^18,24–28^; however several studies made use of ADSCs^29,30,35^. All of them found that BMMSCs or ADSCs accelerate corneal wound recovery by inducing corneal epithelium migration and proliferation. Notably, in these MSC studies, topical administration of CM derived from MSCs was rarely chosen as an MSC administration method for damaged corneas. Most of the experiments selected direct limbal or subconjunctival injection of MSCs, which are most commonly used in in vivo animal studies^24–28,30,35^. In the clinic, most ophthalmologists use topical administration for corneal disease because it is a quick, easy, non-invasive approach. Three studies elected to use topical administration for corneal damage^15,29,30^. None of them used cell-free CM but instead, a solution containing adipose tissue or BMSCs topically.

Corneal epithelialization was significantly accelerated at 2 days following induction of chemical burn in our study. Lin et al. also showed that topical, orbital fat-derived MSCs significantly promoted corneal epithelization at 2 and 3 days after corneal injury compared to the control group^29^. Zeppieri et al., in another topical ADSC study, showed that corneal epithelial defect size decreased significantly from the first days after topical ADSC administration^30^. The difference in epithelial recovery rates could be caused by a difference in corneal defect size. Lin et al.’s and our work applied total epithelial removal after chemical treatment, while Zeppieri et al. used corneal defect induced by laser spots. The corneal epithelium was removed totally in this study, and the pattern of corneal epithelial regeneration was similar to Lin et al.’s study. In this respect, topical cell-free CM employed in this study had an equivalent effect on corneal epithelial regeneration compared to a topical solution containing MSCs.

Regarding the upregulated cytokines in CM-treated corneas, HGF and EGF were significantly overexpressed within the cornea in the CT group compared with those in the CB and NC groups in this study. It has been reported that HGF acts on monocytes, promotes migration, and induces inflammatory cytokine secretion^36^. However, recent studies have found that HGF inhibits TNF-α, IL-1β, and IL-6, and the infiltration of inflammatory cells, resulting in an immune-modulatory effect^37–39^ mediated by an increase in GSK3b phosphorylation and prolonged retention of the phosphorylated p65 subunit in the cytoplasm^39^. Omoto et al. reported that HGF promotes proliferation of the corneal epithelium under homeostatic conditions and reverses the epithelial proliferation inhibitory effect of inflammation^37^. HGF has been reported to have an anti-agonistic effect in the case of increased VEGF expression^40^. MMP9 is also inhibited by HGF because it is induced by inflammatory cytokines such as IL-1β^41,42^. The increases in IL-1β, VEGF, IL-6, and MMP9 levels were significantly less in the CT group than in the CB group during 1 to 3 days after the injury, which is also consistent with a recent study on HGF^37^. EGF expression was significantly elevated in the CT group compared with that in the CB group at 2 and 3 days after injury. EGF is one of the growth factors that plays an important role in corneal epithelial self-renewal and wound healing^43^. Based on the ELISA results in the present investigation, CM contained a low concentration of EGF; corneal tissue treated with CM showed significantly increased indigenous EGF mRNA level compared with the CB group. Particular factors contained in CM might upregulate EGF in chemically damaged corneas, potentially contributing to one of the core functions of the process of re-epithelization. EGFR ligands such as amphiregulin and TGFα were upregulated by IL-6 via an EGFR-dependent pathway of autocrine stimulation in human cervical carcinoma cells and virus-immortalized cells^44^. IL-6, an abundant cytokine in CM, might stimulate EGF/EGFR autocrine secretion in the damaged cornea after topical administration of CM^5,44^. IL-6 is also known as a facilitator for corneal epithelium^45–47^. Nishida et al. reported that IL-6 promotes the migration of corneal epithelial cells both in vitro and in vivo by a fibronectin-dependent mechanism^46,47^. Arranz-Valsero et al. reported that IL-6 is involved in the rapid recovery of corneal epithelium, using an in vitro scratch assay^48^. However, IL-6 mRNA levels were significantly lower in CM-treated corneas than in the CB and NC groups 2 days after injury, and IL-6R levels exhibited no statistically significant difference between the CT and CB groups.

VEGF, MMP9, IL-1β, and IL-6 concentrations were all significantly lower in the CT group than the control group. VEGF is associated with neovascularization following corneal injury^49,50^, and MMP9 is known to be secreted by inflammatory cytokines, such as IL-1β and TNF-α, and is associated with corneal ulcers^41,42^. IL-1β and IL-6 are well known as inflammatory cytokines of the cornea. Inhibition of IL-1β decreases corneal inflammation and increases transparency^51^. Although IL-6 is an inflammatory cytokine, it has been shown to help repair corneal damage^46,47^. In the previous studies, suppression of inflammatory cytokines released after corneal damage showed beneficial effects on improvement of corneal transparency but harmful impacts on corneal epithelial wound healing during the early phase^51^. Contrary to previous observations, even though inflammatory cytokines were suppressed, epithelial corneal defects caused by chemical burn recovered rapidly in the CT group in this study.

In terms of histology, CM-treated corneas showed more PCNA- and ZO-1-stained cells than the NC or CB groups. The levels of ZO-1, which is related to the tight junction of the epithelium, and PCNA, whose expression indicates cell proliferation, were increased in the CT group. It was also histologically observed that CM was effective for proliferation and maturation of the corneal epithelium. Inflammation is an indispensable factor for wound healing, but if inflammation-mediated cytokines are constantly released and inflammation persists, it may interfere with the wound healing process and lead to unexpected results, such as scarring, ulceration, and perforation^52^. In this respect, CM administration appears to exhibit a dual effect that inhibits inflammation and promotes proliferation as well as differentiation of corneal epithelial cells to achieve optimal recovery of the wound by varied cytokine harmony. Omoto et al. reported that the Ki-67 nuclear marker, which is related to cell proliferation, was highly expressed in damaged corneas in the presence of topical HGF, and the HGF receptor was also abundantly expressed, suggesting that HGF promotes epithelial proliferation upon corneal injury^37^. In the current work, CM derived from ADSCs contained a very high HGF concentration. As such, the dual effects of HGF in CM, which could proliferate and migrate corneal epithelial cells as well as suppress inflammation, may contribute to regenerating the corneal epithelium.

One question that remains is why the number of macrophages stained for Iba-1 in the corneal limbus was higher in the CT group than in the CB group. Omoto et al. reported that HGF inhibited the infiltration of cells positive for CD45, which is a leukocyte marker^37^. Despite CM dropping with high concentrations of HGF and the upregulation of corneal HGF mRNA in this study, the number of Iba-1 + cells was significantly increased in the CT group compared with that in the CB group on the first day of injury. We also investigated polarization of corneal-infiltrated macrophages at the limbus into M2. Among the infiltrated Iba-1 + cells, some were co-localized with Iba-1 and arginase-1, and the number of polarized macrophages was higher than that in the CB group in our study (Supplementary Figure). A couple of factors contained in CM could affect macrophage polarization toward M2^53–55^. Further, M2 polarization of macrophages in damaged tissue when administered CM derived from ADSCs has been reported in previous studies^55,56^. In these studies, IL-6 and TSG-6 were shown to polarize monocyte differentiation toward M2^27,57,58^. TSG-6 produced in response to signals from injured tissue displayed remarkable therapeutic effects in the eye and inflammatory bowel disease through macrophage M2 polarization^59^. IL-6-induced IL-4 receptor expression has been seen to augment the response to IL-4 in macrophages in a cell-autonomous manner^60^. These factors could affect the polarization of limbal macrophages in the CT group in this study. Here, CM harvested from ADSCs that were not stimulated with inflammatory mediators contained little TSG-6. In this context, IL-6 might be the central molecule that impacts polarization of macrophages infiltrating the corneal limbus of the CT group.

To evaluate the optimal concentration of CM for topical drops, various concentrations of CM were tested in HCECs in vitro. Interestingly, the lower concentration of CM (× 0.5) had a higher migration rate than that of the higher concentration. IL-6 and CXCR4 mRNAs were upregulated in CMx0.5, which could be involved in epithelial migration. HGF and EGF mRNAs in HCECs were upregulated upon CM treatment, which reflected the previous results of corneal mRNA expression in the CT group. Considering the migration rate differed depending on the CM concentration, optimal concentrations of CM should be evaluated before clinical application as topical drops. The concentration of CM for topical application might be higher because the retention volume and time of CM on the ocular surface can be relatively poor and short, respectively.

Various tissues, such as BM, adipose tissue^6^, cardiac tissue^7^, cord blood^8^, oral tissue^9^, and in particular, corneal limbus, can serve as sources of MSCs^61,62^. As such, corneal limbus could be applied in regenerational medicine. However, the major disadvantage of limbal MSCs is that they are not as abundant as in the case of BM or adipose tissue. For example, some concerns exist regarding potential donor eye risks when harvesting more than 50% of the corneal limbus of one eye, owing to limbal stem cell deficiency^63^. Another disadvantage is the lack of previous research on limbal MSCs compared with research on MSCs derived from adipose tissue or BM. ADSCs are abundant in the human body, making them a potential material for wound healing and tissue engineering with low risks in terms of cell acquisition and easy processing^33^.

In conclusion, CM harvested from ADSCs accelerated recovery from corneal epithelial damage due to chemical injury through the effects of several growth factors. CM can affect proliferation and migration of corneal epithelial cells, reduce inflammatory cytokine levels, and polarize infiltrating macrophages toward M2. Multimodal pathways were involved in the regeneration of the corneal epithelium in this study. In addition, we identified a culture environment for greater secretion of HGF and IL-6 from ADSCs. Further studies on how various growth factors and cytokines interact to affect corneal epithelial regeneration and reveal signal transduction pathways could form the cornerstone of a new corneal regeneration technique.

## Materials and methods

### Research design

Male Sprague–Dawley rats (200–250 g, 6-weeks old, Hyochang Science Inc., Daegu, Korea) were divided into four groups of 10 rats each: a normal control (NC) group, a corneal burn (CB) group, a media control (MC) group, and a CM-treated (CT) group. The NC group did not have any damage and the CB group did not have any intervention after induction of chemical burn. The CT and MC groups were administered topical CM or media (DMEM/F12) after induction of chemical burn, respectively. Topical administration was performed four times a day for 3 days. After finishing evaluation of corneal defect recovery experiments, we compared the CT, CB, and NC groups in a gene and protein study. Corneas, including the limbus, were sampled immediately before injury, 1 day, 2 days, and 3 days after injury for histological and genetic study. Biomicrophotographs were taken using fluorescence staining to assess the degree of corneal damage at the same time points. The study was approved by the Institutional Review Board and Institutional Animal Care and Use Committee of Kosin University College of Medicine (KMAP-16-21). All experiments that involved animal subjects were performed in accordance with the guidelines and regulations of the committee.

### Corneal burn animal model

The animals were anesthetized using 10 mg/kg Zoletil 50 (Virbac, Carros, France) injected intraperitoneally. To induce chemical damage to the cornea, a Whatman #2 filter paper (Whatman, Maidstone, England) with a diameter of 3 mm was soaked in 100% alcohol and applied to the rat cornea for 50 s. The cornea was then rinsed three times using phosphate-buffered saline (PBS). To confirm total de-epithelization, a 0.1% fluorescence solution (Sigma, St. Louis, USA) was instilled and the cornea was photographed. CM derived from rat ADSCs was administered upon removal of the epithelium and then every 6 h for 3 days. Fluorescence images (Motic Images Plus 2.0) were taken using a GFP filter with a stereoscopic microscope (NexiusZoom; JENCO, Milwaukie, USA) equipped with a fluorescence adapter (NightSea, Lexington, USA) before, immediately after, and 24, 48, and 72 h after injury. All procedures were performed by one skilled ophthalmologist. The corneal epithelium defect size was quantified as corneal defect size at each time point/corneal defect size immediately after damage × 100 using a photographed image analyzer (Image Pro Plus; Media Cybernetics Inc., Rockville, USA).

### Culture of ADSCs and harvest of CM

Rat ADSCs were isolated and cultured according to the method described in a previous study (Heo et al.^64^). Briefly, adipose tissue was obtained from rat epididymal and subcutaneous fat and processed to obtain a stromal vascular fraction (SVF). The adipose tissue was washed 2–3 times using PBS (Lonza, Basel, Switzerland) for cell substrate fractionation. The extracellular matrix was treated with 0.075% type 1 collagenase (Worthington, Lakewood, USA) in PBS for 30 min in a 37 °C water bath. The enzyme activity was neutralized with Dulbecco's modified Eagle's medium: Nutrient mixture F-12 (DMEM/F12; Gibco BRL, Grand Island, USA) containing 10% FBS (HyClone, Logan, USA), and centrifuged at 1,200 g for 10 min to obtain a high density of cell substrate fraction pellets. The cell pellet was resuspended in DMEM/F12 and filtered through a 40-μm mesh to remove cell debris. Cells were then cultured in a basic culture medium containing DMEM/F12, 10% FBS, and 1% penicillin/streptomycin solution (Gibco BRL) at 5% CO_2_ in an incubator. After culturing, the cells were washed 2–3 times using PBS to remove residual unattached red blood cells. To prevent spontaneous differentiation, ADSCs were maintained at subconfluence. The cells were subcultured at a 1:4 ratio by treatment with 0.05% trypsin (Gibco BRL) for 5 to 10 min. Three to seven subcultured stem cells were used in all the experiments.

To harvest CM, rat ADSCs from passage 4–5 were seeded onto a culture dish (100 mm) at 1 × 10^6^ cellular concentration and cultured in DMEM/F12 with EGF and bFGF until 80–90% confluence. After removing the culture media, the cells were washed twice using PBS and the medium was replaced with 5 ml of DMEM without serum or growth factors. The CM was collected after culturing for 24 h and concentrated to 60 × using Amicon Ultra-15 Centrifugal Filter Units (Millipore, Burlington, USA). The concentrated CM was used as topical eye drops after dilution to 30 × in DMEM/F12.

### Characterization of rat ADSCs

For flow cytometry, P3 rat ADSCs (passage 3) were treated with 0.05% trypsin (Gibco BRL) and resuspended in PBS containing 1% bovine serum albumin (BSA). They were then treated with antibodies for 30 min in a dark room at room temperature. Fluorescein isothiocyanate (FITC)-conjugated antibodies were used for the analysis of CD31 (Abcam, Cambridge, UK), CD34, CD105, CD73 (Bioss Antibodies, Woburn, USA), CD45, and CD90 (BD Biosciences, San Jose, USA). Cells were analyzed using a flow cytometer (BD Accuri C6 Plus Flow Cytometer; BD Biosciences) and the data were analyzed using BD Accuri C6 Software (BD Biosciences).

In vitro differentiation of rat ADSCs was performed in the same manner as previously reported^64^. ADSCs were cultured 3 to 7 times and recovered by treatment with 0.05% trypsin (Gibco BRL). The cells were counted using 0.4% trypan blue (Gibco BRL) staining and dispensed into 12-well plates at 1 × 10^4 ^cells/cm^2^. Differentiation was induced using adipogenic differentiation medium (A1007001; Gibco BRL), chondrogenic differentiation medium (A1007101; Gibco BRL), and osteogenic differentiation medium (A1007201; Gibco BRL). The differentiation medium was changed once every 3 to 4 days. After differentiation into adipose, bone, and cartilage, the cells were stained with Oil Red O (Sigma), Alizarin Red S (Sigma), and Alcian Blue (Sigma), respectively.

### Migration and proliferation assay

To measure migration capacity depending on culture conditions, rat ADSCs passaged 3 to 5 times were recovered by treatment with 0.05% trypsin (Gibco BRL). Cells were counted using 0.4% trypan blue (Gibco BRL) staining and dispensed into 6-well plates at 0.5 × 10^4 ^cells/cm^2^. When cell density was 100%, scratches were made at a size of roughly 0.4–0.5-mm width using a 200-ml universal pipette tip (Neptune Scientific, San Diego, USA), and then washed twice using PBS to remove the remaining cells. Culture medium was composed of various combinations of DMEM or DMEM/F12 with two kinds of growth factors (bFGF, EGF) for a total of eight types: 10% DMEM, 10% DMEM + bFGF, 10% DMEM + EGF, 10% DMEM + bFGF + EGF, 10% DMEM/F12, DMEM/F12 + bFGF, DMEM/F12 + EGF, and DMEM/F12 + bFGF + EGF. The growth factors were recombinant rat proteins (Peprotech, USA). The eight kinds of culture media (2 ml) were added and photographed at the same place using a phase contrast microscope (Nicon-Eclipse, Nikon, Japan) at 0 h and 24 h. The photographs of migration were taken at 100 × magnification and the area of the empty space into which the cells migrated was measured. The % value of the remaining area was calculated using the wound area (24 h)/wound area (0 h) × 100, and the value of the area occupied by the cells was calculated by subtracting 100 from the resultant value.

In order to measure the proliferative capacity of rat ADSCs according to the composition of the medium, the cells were cultured in a 100-mm culture dish with each constituent medium. Cells (passage 5) were seeded at a density of 1 × 10^4 ^cells/cm^2^ on a 24-well plate. At 2 days and 4 days of incubation, the degree of cell viability was measured using an MTT [3-(4,5-dimethylthiazol-2yl)-2,5-diphenyltetrazoliumbromide] assay at 540 nm. The MTT (Cyto X, LPS solution, Daegu, Korea) reagent was added to the cultured cells, which were then incubated at 37 °C for 2 h. The amount of MTT degradation to formazan was determined by measuring the absorbance at 540 nm using a spectrophotometer (EMax; Molecular Devices, San Jose, USA). Proliferative capacity was examined by comparing the average values for the experimental groups according to the composition of the medium.

### Enzyme-linked immunosorbent assay (ELISA)

The amount of protein secreted by rat ADSCs was determined by ELISA according to the composition of eight different media. The CM harvesting method was the same as that used for topical CM formation. Each medium collected after culturing for 24 h in the serum-free culture medium was used as a protein analysis sample for ELISA. The media were diluted to 30 × and used for analysis. EGF, TGFβ1, and HGF proteins were analyzed by ELISA (HGF and EGF; R&D Systems, Minneapolis, USA; TGFβ1; Abcam, Cambridge, UK). A total of 100 μl of each of the pre-enrichment culture medium and standard solution for the protein was added to the wells coated with the primary antibody followed by reaction at 37 °C for 2 h. After the reaction was completed, the solution contained in the well was removed. Then, 100 μl of the secondary antibody (polyclonal antibody specific for mouse/rat EGF, TGFβ1, and HGF conjugated to horseradish peroxidase with preservatives) was added at 37 °C for one hour. After completion of the secondary antibody reaction, each well was washed three times, 90 μl of the substrate solution was added, and the reaction was carried out at room temperature for 30 min with light blocked. Each cytokine concentration was measured using a spectrophotometer (EMax; Molecular Devices) at 490 nm. Normal medium was used for baseline activity comparisons.

### Quantitative real-time polymerase chain reaction (RT-qPCR)

The amount of mRNA expressed by rat ADSCs was determined according to the composition of the eight different media. P5 rat ADSCs at a density of 90% cultured on a 100-mm culture dish with each medium composition were washed twice using PBS and replaced with 5 ml of DMEM/F12 containing no serum or growth factors. Each medium collected after culturing for 24 h was used for mRNA extraction. Transforming growth factor β 1 (TGFβ1), vascular endothelial growth factor (VEGF), nerve growth factor (NGF), epidermal growth factor (EGF), interleukin-6 (IL-6), insulin-like growth factor (IGF-1), hepatocyte growth factor (HGF), fibroblast growth factor 7 (FGF7), matrix metallopeptidase-9 (MMP-9), α-smooth muscle actin (αSMA), interleukin-1β (IL-1β), interleukin-6 receptor (IL-6R), and interleukin-10 (IL-10) of rat cornea and ADSCs were analyzed by real-time PCR. Cytokeratin 3, MMP-1, MMP-3, IL-6, EGF, HGF, cyclin-dependent kinase (CDK) 1, CDK2, and C-X-C chemokine receptor type (CXCR) 4 expression in HCEC were analyzed by RT-PCR. Three or more corneas, including the limbus detached from the eyeballs, in each group at 0, 24, 36, and 48 h after chemical corneal epithelial injury, were employed for assessment of rat ADSCs and HCECs were used for gene analysis. Total RNA was extracted using TRIzol (Invitrogen, Carlsbad, USA) and the concentration was measured using a Nanodrop (NanoDrop 1,000; Thermo Fisher, Waltham, USA). Total RNA (3 μg) was reverse transcribed into cDNA using a TOPscript cDNA synthesis kit (Enzynomics, Daejeon, Korea). RT-qPCR was performed to determine the level of mRNA expression using SYBR Green PCR Core Reagents kit (Applied Biosystems). The sequences of the PCR primers are shown in Table 1. The following conditions were used: 95 °C/15 min, followed by 40 cycles of 95 °C/30 s, 60 °C/30 s, and 72 °C/30 s in the 7,300 Real-time PCR apparatus (Applied Biosystems). The levels of mRNA expression were normalized to that of the internal control housekeeping gene, GAPDH. Relative levels of mRNA expression were compared against control.

**Table 1:** Primer sequences used for gene expression analysis by RT-PCR.

Species	Gene	Forward	Reverse
Rat	HGF	AAGGTTACAGGGGAACCACC	AGAGCAGTAACCAACTCGGA
Rat	TGFβ	TGGAAAGGGCTCAACACCTG	AGAAGTTGGCATGGTAGCCC
Rat	FGF7	TGTCTTGTGGGCACCATATCT	AACTTCTCGTGTGTCGCTCG
Rat	NGF	GTGCCCCTGCTGAACCAATA	GTCCGTGGCTGTGGTCTTAT
Rat	EGF	GTCGTACGATGGGTACTGCCTC	GCGCAGCTTCCACCAACGTAAG
Rat	VEGF	ACTTGACTTCTGTTGCCTCG	TCCCAGGGGTAGCTGTAAAGT
Rat	MMP-9	AGCTACACCGAAGACTTGCC	GAAAGGCGTGTGCCAGTAGA
Rat	αSMA	TCATTGGAATGGAGTCGGCG	TGCGTTCTGGAGGAGCAATAAT
Rat	IL-1β	ATGAGGACCCAAGCACCTTC	AGCTCACATGGGTCAGACAG
Rat	IL-6	CTGGTCTTCTGGAGTTCCGTT	GGTCTTGGTCCTTAGCCACTC
Rat	IL-6R	ACGTTTCCTGTCCCCCTACT	TTGTAAAAGGCAAGCTCCT
Rat	IL-10	CAGCAAAGGCCATTCCATCC	TTGGCAACCCAAGTAACCCT
Rat	IGF1	CTGGTGGACGCTCTTCAGTT	CTTCAGCGGAGCACAGTACA
Rat	GAPDH	GAAGCTGGTCATCAACGGGA	GAAGGGGCGGAGATGATGAC
Human	MMP1	CTTCCCAGCGACTCTAGAAACA	TGCTTCATCACCTTCAGGGTT
Human	MMP3	GGTTCCGCCTGTCTCAAGAT	AGGGATTTGCGCCAAAAGTG
Human	MMP9	GGTGATTGACGACGCCTTTG	GAAATGGGCGTCTCCCTGAA
Human	IL-6	AGCATCCCTCCACTGCAAAG	GTGCCCATGCTACATTTGCC
Human	EGF	TTCTACTTGTGTGGGTCCTGC	ACTCTCTCTTGCCTTGACCC
Human	HGF	CAATGCCTCTGGTTCCCCTT	AGCTCGAAGGCAAAAAGCTG
Human	CDK1	GCACTTGGCTTCAAAGCTGG	CATGGCTACCACTTGACCTGT
Human	CDK2	GCATCTTTGCTGAGATGGTGAC	GCTTGTTAGGGTCGTAGTGC
Human	CXCR4	TCACTATGGGAAAAGATGGGGA	GAGCCCATTTCCTCGGTGTA
Human	BETA ACTIN	GGCATCGTCACCAACTGGGAC	CGATTTCCCGCTCGGCCGTGG

Standard reverse-transcription PCR was used for the primers, such as MMP1, MMP3, MMP9, IL-6, EGF, HGF, CDK1, CDK2, and CXCR4 of HCECs. PCR was performed with TB Green Premix Ex Taq II (Takara, Japan) in 20 µl containing 2 µl of cDNA, 0.25 µM concentration of primer, and 10 µl of the 2 × PCR Master Mix. The mixture was subjected to 35 cycles of 15 s at 95 °C for denaturation and 30 s at 60 °C for primer annealing and extension. The PCR products were analyzed using agarose gel electrophoresis.

### Immunohistochemistry

The rats were sacrificed at 0, 24, 36, and 48 h after induction of chemical injury, and three or more cornea containing the corneal limbus were separated from the eyeballs in each group. For the preparation of frozen sections, the collected corneas were fixed with 4% PFA and then incubated at 4 °C overnight. The tissue was dehydrated in 30% sucrose for approximately 1 day and embedded in an OCT compound to create a block. For paraffin sections, the collected tissues were fixed with 4% formalin, dehydrated in alcohol, embedded in paraffin, and made into blocks. For hematoxylin–eosin (HE) staining and immunofluorescence staining, 5 μm of paraffin block was excised. The paraffin slides were washed using Xylene (Duksan, Ansan, Korea) for 5 min to deparaffinize them and then washed for 2 min each with decreasing concentrations of ethanol (100%, 95%, 80%, 70%) (Duksan). Then, for antigen retrieval, the paraffin slides were immersed in 1X citrate buffer (pH 6.0; Sigma-Aldrich) and heated for 2 min in a microwave oven. For immunostaining, the slides was pretreated at room temperature for 1 h in a solution of 4% goat serum and 0.5% Tween-20 in PBS. Treatment with primary antibodies was then performed at 4 °C for 1 h. After the reaction, the slider was washed using PBS three times for 5 min and then treated with secondary antibody. The secondary antibody was treated at room temperature for 1 h, washed again using PBS three times for 5 min, and observed with a fluorescence microscope (NIKON-E600-FL; Nikon, Tokyo, Japan). Proliferating cell nuclear antigen (PCNA; BD Biosciences), ionized calcium-binding adaptor molecule-1 (Iba-1; Wako, Richmond, USA), zona occludens-1 (ZO-1; Thermo Fisher Scientific), and arginase-1 (Novus Biologicals, Centennial, USA) were used as the primary antibodies. The secondary antibodies were goat anti-Mouse Alexa Fluor 568 and goat anti-Rabbit Alexa Fluor 568 (Life Technologies, USA). The sections were then stained with 4′, 6-diamidino-2-phenylindole (DAPI, Sigma-Aldrich) and washed three times using PBS. The slides were shaken and covered with a cover slip each after dropping 2 to 3 drops of a water-soluble sealant (Crystal/Mount; Biomeda, Burlingame, USA) and stored at 4 °C in a refrigerator or at − 70 °C for a prolonged period. The number of PCNA-, Iba-1-, ZO-1-, and arginase-1-positive cells in the corneal center and limbus were counted. The fluorescence of the secondary antibody bound to the primary antibody and DAPI was separately photographed (400 ×) in three different portions of immunohistochemical staining tissue sections. The number of cells positive for staining contained in a specific fraction (134 μm × 56 μm) of the picture was counted, but the determination of staining positive was defined as the case where immunofluorescence was expressed in more than 50% of the cell size.. ZO-1, Iba-1, and arginase-1 were analyzed in the same manner.

### Human corneal epithelial cells (HCECs)

HCECs were purchased from ATCC and cultured according to the company’s instructions. Growth medium for HCECs was prepared using supplemented Keratinocyte SFM (Gibco, USA) by adding the entire contents of the BPE and EGF supplement vials. Frozen cell vials were thawed, and the cells were seeded at 5 × 10^3 ^cells/cm^2^. Cultures were incubated at 37 °C under 95% humidity and 5% CO_2_. The medium was changed every 24 to 48 h. To confirm that the cells were HCECs, the genes for epithelial cell marker cytokeratin 3 (Krt14, 191 bp) were amplified by RT-PCR. The HCEC migration assay was similar to the rat ADSC assay except for the culture media composition and time points of observation. Culture medium for the HCEC migration assay was composed of HCEC medium containing supplements with various concentrations of CM (× 0.5, × 1.0, × 3.0, × 5) derived from ADSC culture. The time points for migration were 0 and 36 h. To measure the proliferative capacity of HCECs, the cells were cultured in a 60-mm culture dish for each constituent medium, which had the same composition as the medium used in the migration assay. HCECs were seeded at a density of 1 × 10^4 ^cells/cm^2^. After 2 days of incubation, the number of living cells was counted using a hemocytometer following 0.1% trypan blue staining.

### Statistical analysis

Data are expressed as mean ± standard deviation. One-way analysis of variance (ANOVA) or repeated-measures two-way ANOVA were performed using GraphPad prism 7 (GraphPad, San Diego, USA). A *p*-value < 0.05 was considered statistically significant. Significance is highlighted as follows: *p < 0.05; **p < 0.01; ***p < 0.001; ****p < 0.0001. In vivo animal experiments were repeated five times and in vitro experiments three times to increase reliability.

## Supplementary information

Supplementary Information.
