# Supplementary material for: Topical cell-free conditioned media harvested from adipose tissue-derived stem cells promote recovery from corneal epithelial defects caused by chemical burns
Source: Sci Rep. 2020 Jul 24;10:12448. doi: 10.1038/s41598-020-69020-z (PMC7381646; doi:10.1038/s41598-020-69020-z)

**Topical Cell-free Conditioned Media Harvested from Adipose Tissue-derived Stem Cells Promote Recovery of Corneal Epithelial Defect Caused by Chemical Burn**

Kae Won Park^1^, Jeonghoon Heo^2^, Jung Yup Kang^3^, Ji Won Yang^1,4^, Jong Sik Kim^5^, Ki Dong Kwon^4^, Byung Chul Yu^6^, Sang Joon Lee^4^

Supplementary information.


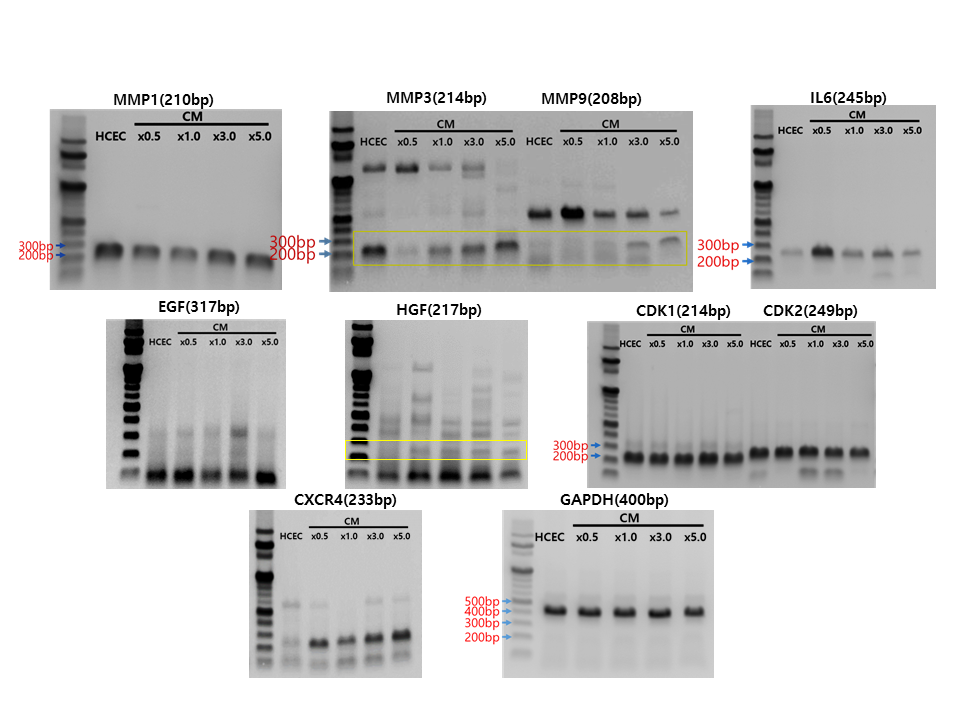


Supplementary figure 1. Immunohistochemistry of Arginase-1 and Iba-1 in cornea harvested from cell-free conditioned media treated (CT) group (A~D), corneal burn (CB) group (E~F), and normal control (NC) group (H~K). CT groups showed much more Arginase-1 and Iba-1 stained cells in the cornea than that of CB and NC groups (white arrows).


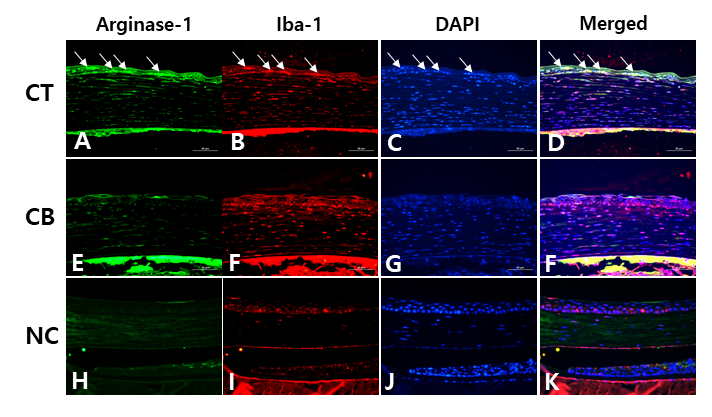

Supplement: Supplementary file 1 — Supplementary Information. [file 41598_2020_69020_MOESM1_ESM.docx]
